# DYRK1A inhibition restores pancreatic functions and improves glucose metabolism in a preclinical model of type 2 diabetes

**DOI:** 10.1016/j.molmet.2025.102242

**Published:** 2025-08-29

**Authors:** Romane Bertrand, Stefania Tolu, Delphine Picot, Cécile Tourrel-Cuzin, Ayoub Ouahab, Julien Dairou, Emmanuel Deau, Mattias F. Lindberg, Laurent Meijer, Jamileh Movassat, Benjamin Uzan

**Affiliations:** 1 Université Paris Cité, BFA, UMR 8251, CNRS, Team « Endocrinology of Diabetes and Fertility », F-75013 Paris, France; 2 Université Paris Cité, UMR 8601 CNRS, Laboratoire de Chimie et Biochimie Pharmacologiques et Toxicologiques, 75006 Paris, France; 3 Perha Pharmaceuticals, Hôtel de Recherche, Presqu’île de Perharidy, 29680 Roscoff, France

**Keywords:** Type 2 diabetes, β cell proliferation, Dyrk1A inhibitors, Goto-Kakizaki rat, Glucose metabolism, Pancreatic islets

## Abstract

**Objectives:**

Insulin deficiency caused by the loss of β cells and/or impaired insulin secretion is a key factor in the pathogenesis of type 2 diabetes (T2D). The restoration of β cell number and function is thus a promising strategy to combat diabetes. Dual-specificity tyrosine-regulated kinase 1A (DYRK1A) has been shown to regulate human β cell proliferation. DYRK1A inhibitors are potential therapeutic tools, due to their ability to induce β cell proliferation. However, their anti-diabetic effects in the complex setting of type 2 diabetes remains unexplored. The aim of this study was to determine the impact of chronic DYRK1A inhibition on the remission of diabetes in pre-diabetic and overtly diabetic Goto-Kakizaki (GK) rats.

**Methods:**

We assessed the impact of *in vivo* treatment with a DYRK1A inhibitor, Leucettinib-92, on β cell proliferation and insulin secretion in GK rats. Further, we evaluated the effects of long-term Leucettinib-92 treatment on the whole-body glucose metabolism in overtly diabetic GK rats through the assessment of fasting and post-absorptive glycemia, glucose tolerance and insulin sensitivity.

**Results:**

Short-term *in vivo* treatment of prediabetic GK rats with Leucettinb-92 stimulated β cell proliferation *in vivo*, and sustainably prevented the development of overt hyperglycemia. Long-term treatment of adult GK rats with established diabetes increased the β cell mass and reduced basal hyperglycemia. Leucettinib-92 treatment also improved glucose tolerance, and glucose-induced insulin secretion *in vivo*.

**Conclusions:**

We show that DYRK1A inhibition restores the β cell mass and function in a preclinical model of T2D, leading to the improvement of body's global glucose homeostasis.

## Highlights

• DYRK1A is implicated in β cell proliferation.
• DYRK1A inhibition with Leucettinib-92 stimulates the proliferation of β cells in both healthy and diabetic rats.
• Inhibition of DYRK1A with Leucettinib-92 in prediabetic GK rat prevents the development of overt diabetes.
• Inhibition of DYRK1A in GK rats with established diabetes restores the β cell mass and alleviates hyperglycemia.

## 1 Introduction

Diabetes is a fast-growing chronic disease with severe complications. Diabetes is characterized by chronic hyperglycemia, whose origin varies according to the type of diabetes. In type 1 diabetes (T1D) the near total destruction of pancreatic β cells by immune cells and the consequent insulinopenia is the cause of elevated blood glucose levels. Type 2 diabetes (T2D) is a multifactorial disease representing ∼90% of diabetes cases, which results from two distinct but interrelated pathologies: differing degrees of increased insulin resistance and decreased insulin secretion [1,2]. The growing incidence of T2D is mostly driven by insulin resistance and obesity, but the key point is that most people with insulin resistance will never develop T2D. Diabetes only occurs if their β cells fail to provide sufficient insulin [1,2].

Therefore, both types of diabetes are associated with β cell deficiency [3]. The replenishment of β cell population within the pancreas is thus one of the most promising strategies to address diabetes. The identification of molecular pathways and targets to restore the defective β cell mass is of major importance for the development of regenerative therapies for this high impact pathological condition [[4], [5], [6]]. During the last decade, the Dual-specificity tyrosine phosphorylation Regulated Kinase 1A (DYRK1A) has emerged as a target for the induction human β cell proliferation [[7], [8], [9]].

DYRK1A is a member of the DYRK family which comprises five subtypes (1A, 1B, 2, 3, 4) [10,11]. DYRK1A is ubiquitously expressed and has attracted increasing interest as a potential therapeutic target in various diseases such as Alzheimer's disease, Down syndrome and leukemias [8,10,12,13]. Only a decade ago, another facet of DYRK1A was discovered. Several groups have reported that genetic or pharmacological inhibition of DYRK1A leads to β cell proliferation in human and rodent islets, raising the interest in its potential application to diabetes [[11], [12], [13], [14], [15]]. Current understanding of the various mechanisms underlying the regulation of β cell proliferation by DYRK1A, implicates its interaction with the nuclear factor of activated T cells (NFAT) family of transcription factors. Nuclear NFAT transactivates promoters of cell cycle activating genes and represses promoters of cell cycle inhibitory genes, resulting in cell cycle entry and proliferation [15]. Phosphorylation of NFATs by nuclear DYRK1A forces their export from the nucleus to the cytoplasm, thus terminating cell proliferation. An additional potential mechanism underlying the regulation of β cell proliferation by DYRK1A involves cyclin D1. DYRK1A phosphorylates cyclin D1 on Thr286, leading to cyclin D1 degradation and cell cycle arrest. Inhibition of DYRK1A leads to cyclin D1 stabilization and accumulation. Accumulation of cyclin D1 may favor pancreatic β cell proliferation [19,20]. Although the pro-mitotic effects of DYRK1A in β cells are now well documented [7], studies in the beneficial effects of DYRK1A inhibition on the whole-body glucose homeostasis are scarce.

In this study, we used a DYRK1A inhibitor from the Leucettinib family: Leucettinib-92 [21]. Leucettinib-92 (LCTB-92) is the most potent DYRK1A inhibitor of the family (with a hydroxy-derivative). The comparison of Leucettinib-92 with other Dyrk1A inhibitor, especially with harmine showed that harmine was a modest DYRK1A and DYRK1B inhibitor compared to Leucettinib-92 [22]. Moreover, harmine inhibits other DYRKs and CLKs and interacts also with CDK8, CDK9 and CSNK1G3 [23]. Harmine is also a very potent inhibitor of monoamine oxidase MAO-A [24,25] and this can contribute to off-target effects of harmine. Therefore, we selected Leucettinib-92 for the present study to evaluate its effects on cell proliferation in isolated islets of healthy Wistar and diabetic Goto-Kakizaki (GK) rats. Moreover, we assessed the effects of long-term pharmacological inhibition of DYRK1A on the evolution of the diabetic status in both pre-diabetic and overtly diabetic GK rats.

We show that Leucettinib-92 has a potent proliferative effect *ex vivo*, in β cells from non-diabetic and diabetic rats. More importantly, DYRK1A inhibition in GK rats efficiently increases β cell mass and secretory function in juvenile and adult rats, leading to the prevention or remission of diabetes.

## 2 Materials and methods

### 2.1 1-INS-1 832/13 β cell culture and treatments

The rat insulinoma β-cell line INS-1 832/13 was used between passages 15 and 20. Cells were cultured at 5% CO_2_ and 95% air at 37 °C in RPMI medium-1640 containing 11 mM of d-Glucose supplemented with 10% heat-inactivated fetal calf serum (Eurobio, Les Ulis, France, ref. CVFSVF00–01), 100 u/mL Penicillin-Streptomycin, 10 mM HEPES, 1 mM Sodium pyruvate, 2 mM l-glutamine and 50 μM β-mercaptoethanol (Invitrogen, Saint Aubin, France).

A dose–response assay was carried out to determine the dose of Leucettinib-92 to be used for the *ex vivo* experiments. INS-1 832/13 cells were seeded in 6-well cell culture plates and treated with either DMSO or with 0.5, 5 or 50 nM Leucettinib-92 for 48h. At the end of the treatment period, cells were counted using the automated cell counter ADAM-MC (NanoEnteck Europe, Martinsried, Germany).

The effect of LCTB-92 used at the concentration of 50 nM on cell growth in INS-1 832/13 β cells was also compared with that of harmine (Fisher Scientific, Illkirsh, France) used at either 50 nM or 10 μM.

Leucettinib-92 was synthetized at Perha Pharmaceuticals as described previously [18].

### 2.2 *Ex vivo* experimentation

Animals used in this study were male Wistar and Goto-Kakizaki (GK) rats from our local colony (GK/Par). Animal housing and breeding and relevant procedures were conducted in accordance with the European Community Council directives (2010/63/UE) and approved by the institutional Animal Care and Use Ethical Committee of the Université Paris Cité (Agreement B-75-13-17, CEB-33-2021). Animals were housed in ventilated cages in controlled environment (temperature = 20 +/− 1 °C; humidity = 60%) and 12 h light/dark cycle, with standard enrichment. Animals had *ad libitum* access to food (diet D113, SAFE, Augy, France) and water.

#### 2.2.1 Islet isolation

Three-month-old Wistar and GK rats were killed by the administration of a lethal dose of pentobarbital. Pancreases were collected and digested with liberase (Roche Diagnostics, Boulogne-Billancourt, France, ref. 05401020001) in Hank's buffer (Sigma Aldrich, Saint Quentin Fallavier, France, ref. H8264) for 15 min (Wistar) or 17 min (GK) in a 37 °C water bath. After 4 washes with Hank's buffer, islets were hand-picked under a stereomicroscope. Collected islets were either immediately used to measure glucose-induced insulin secretion or cultured for further experiments.

#### 2.2.2 Measurement of glucose-induced insulin secretion

Ten sized-matched islets were stabilized by pre-incubation at 37 °C in a humidified atmosphere for 1 h in 1 mL Krebs–Ringer/bicarbonate/Hepes buffer (115 mM NaCl, 5 mM KCl, 24 mM NaHCO_3_, 1 mM CaCl_2_, 1 mM MgCl_2_) containing 0.2% fatty-acid-free BSA (Roche, Boulogne-Billancourt, France, ref. 10775835001) and 2.8 mM glucose, followed by 1 h incubation in 1 mL of buffer containing 2.8 or 16.7 mM glucose. Supernatants were collected and stored at -20 °C to measure glucose-induced insulin secretion.

Insulin released by islets was normalized to islet's DNA content.

#### 2.2.3 Islet culture and treatments

Batches of freshly isolated islets were cultured in RPMI 1640 + Glutamax medium (Gibco, Illkirch, France, ref. 61870036) with 10% fetal calf serum (Eurobio, Les Ulis, France, ref. CVFSVF00–01), 1% Penicillin-Streptomycin (Sigma, Saint Quentin Fallavier, France, ref. P4333), 1% Hepes (Sigma, Saint Quentin Fallavier, France, ref. H0887), 1% Sodium pyruvate (Sigma, Saint Quentin Fallavier, France, ref. S8636) during 36 h. Leucettinib-92 was dissolved in dimethyl sulfoxide (DMSO). Islets were then treated either with Leucettinib-92 (50 nM) or DMSO (Sigma Aldrich, Saint Quentin Fallavier, France, ref. D8418) for 48 h. Other batches of islets were treated with harmine (Fisher Scientific, Illkirsh, France) at the concentration of 10 μM. Eight hours before the end of the experiment, islets were dissociated for 5 min with trypsin (Trypsin–EDTA (0.05%), Gibco, Canada, ref.25300054) at 37 °C and dissociated cells were cultured in the same medium as described above, supplemented with Leucettinib-92 (50 nM) or with DMSO. After 8 h culture, cells were fixed in 4% paraformaldehyde for 20 min, then washed with PBS and kept at 4 °C until processed for immunofluorescence staining.

#### 2.2.4 Immunofluorescence staining

Following treatments and fixation, islet cells were permeabilized with Triton 0.2% for 15 min and incubated with 10% goat serum for 1 h. Cells were then incubated with anti-Ki67 antibody for 1 h (Abcam, Amsterdam, Netherlands, ref. ab16667) followed by incubation with DyLight 549-conjugated anti-rabbit secondary antibody (VectorLabs, Newark, USA, ref. DI-1549) for 1 h at room temperature. Cells were then incubated with anti-insulin primary antibody (Santa Cruz Biotechnology, Heidelberg, Germany, ref. sc-8033) overnight at 4 °C, followed by incubation with DyLight 488-conjugated anti-mouse secondary antibody (Sera Care, USA, ref. 5230-0391) for 1 h at room temperature. Finally, cells were covered with mounting medium containing DAPI. Images were acquired using a BX60F5 Olympus microscope. Results were expressed as the percentage of double Ki67/insulin-positive cells over total insulin-positive cells.

#### 2.2.5 siRNA mediated downregulation of Dyrk1A

Islets were isolated from three-month-old Wistar rats. Islets from each pancreas were dissociated as described above and transfected using the HiPerFect Transfection Reagent (Qiagen, pays, ref. 301704). Anti-DYRK1A (5′-CAAGAATGGGTCGCCATTAAA-3′) and control siRNAs (siCTL) (5′-AATTCTCCGAACGTGTCACGT-3′) were prepared in antibiotics and serum-free OPTI-MEM + Glutamax medium (Gibco, Illkirch, France, ref. 51985-026) medium according to manufacturer's instructions. Cells were cultured in OPTI-MEM medium supplemented with 10% fetal calf serum, 1% Penicillin-Streptomycin, 1% Hepes, 1% Sodium pyruvate, and supplemented with 5 nM DYRK1A siRNA (Qiagen, Courtaboeuf, France, ref. 1027417) or control siRNA 5 nM (Qiagen, Courtaboeuf, France, ref. 1022076). After 48 h culture, cells were either directly collected for western blot analysis or fixed and used for Ki67 immunofluorescence staining as described above.

#### 2.2.6 Western blot analysis

Western blot analysis was performed in rat islets. Islets were lysed in RIPA buffer (Cell Signaling, Saint Quentin Fallavier, France, ref. 9806) with 4% protease inhibitor cocktail PIC, PIC2, PIC3 (Sigma, Saint Quentin Fallavier, France, ref. P5726). Fifteen μg protein were subjected to SDS-PAGE and transferred on a PVDF membrane (Sigma, Saint Quentin Fallavier, France, ref. IPVH09120). After blocking with 5% fatty acid free BSA, the membrane was incubated with a mouse anti-DYRK1A primary antibody (Clinisciences, Nanterre, France, ref. H00001859-M01). After incubation with the appropriate horseradish peroxidase-conjugated secondary antibody (Jackson Labs, Las Vegas, USA), the reaction was visualized using the chemoluminescence system ECL Prime (Amersham, Les Ulis, France, ref. 29018903). Membranes were then stripped using Re-Blot solution (Millipore, Molsheim, France, ref. 2504) and incubated with anti-β actin antibody (Sigma Aldrich, Saint Quentin Fallavier, France, ref. A5441) for 1 h, followed by the incubation with the appropriate horseradish peroxidase-conjugated secondary antibody (Jackson Labs, Las Vegas, USA). Blots were analyzed and quantified by scanning densitometry using FIJI®.

### 2.3 *In vivo* experimentation

#### 2.3.1 Experimental designs and treatments

Five-week-old Wistar and GK rats were randomly allocated to 2 groups. This stage of life in the GK model can be considered as a state of “prediabetes”, because animals have already reduced β cell mass and mild hyperglycemia (<200 mg/dL), but are not overtly hyperglycemic. Rats were treated daily (09:00 a.m.) for 5 days by intraperitoneal injection with Leucettinib-92 (0.5 mg/kg body weight). Leucettinib-92 was dissolved in dimethyl sulfoxide (DMSO) and stored at −20 °C. The final formulation was prepared each day just before use, by dilution in saline solution and Cremophor EL (Merck, Burlington, USA, ref. 238470) 95/5 (v/v). The second group was treated with DMSO diluted in saline solution and Cremophor EL 95/5 (v/v) and served as control group. The last injection was done the day of sacrifice. At the end of the treatment, rats were killed by the injection of a lethal dose of pentobarbital, and pancreases were collected. One hour before sacrifice, animals were injected with a solution of 5-Bromo-2′-deoxyuridine (BrdU) (Sigma–Aldrich, Saint Quentin Fallavier, France, ref. B5002) at the dose of 50 mg/kg body weight. Another group of 5-week-old GK rats received the same treatment (Leucettinib-92 or DMSO) as described above. At the end of the 5-day treatment period, animals were maintained alive for five more weeks without treatment. During this period, the body weight and glycemia were monitored once a week. Blood glucose was measured at 09:00 a.m. in blood samples taken from the tip of the tail using an Accu-Check Glucometer (Roche, Boulogne-Billancourt, France). Animals were killed at the end of the fifth week of treatment discontinuation.

In another set of experiments, 3-month-old GK rats with overt diabetes were treated daily for 8 consecutive weeks with either Leucettinib-92 or DMSO (Sigma Aldrich, Saint Quentin Fallavier, France, ref. D8418). Because of the duration of the treatment period (2 months), the dose selected for the chronic treatment was 0.05 mg/kg body weight. During the course of treatment, the body weight and random-fed glycemia (09:00 a.m.) were monitored weekly. One week before the end of the treatment, glycated hemoglobin (HbA1c) was measured by using the Cobas b 101 instrument (Roche Diagnostics, Boulogne Billancourt, France, ref. 08038694190), in blood samples collected at the tip of the tail.

Finally**,** 3-month-old non-diabetic Wistar rats were treated daily for 6 consecutive weeks with either Leucettinib-92 or DMSO. Body weight and glycemia were monitored once a week. At the end of the treatment, rats underwent intraperitoneal glucose tolerance tests (ipGTT) and insulin tolerance test (ipITT) as described below. The pancreatic insulin content was determined by ELISA in pancreases recovered from Leucettinib-92 and DMSO-treated rats, at the end of the experiments.

#### 2.3.2 Body composition

At the end of the treatment period, body composition was determined by a positron emission tomographic whole-body composition analyzer (EchoMRI, Houston, TX, USA).

#### 2.3.3 Biochemical assays

Alanine aminotransferase (ALAT) and aspartate aminotransferase (ASAT) were measured in plasma samples using an Olympus AU400 Automated Clinical Chemistry Analyzer.

The measurement of plasma triglycerides was carried out using the Triglycerides FS∗ Kit (DiaSys, Holzheim, Germany) according to manufacturer's instructions.

#### 2.3.4 Metabolic tests

At the end of treatments, animals were subjected to glucose tolerance tests and insulin tolerance tests.

#### 2.3.5 Glucose tolerance test

After 6-h fasting with free access to water, intraperitoneal glucose tolerance tests (ipGTT) were performed in non-anaesthetized GK rats. Rats received an intraperitoneal injection of glucose at the dose of 1 g/kg body weight. Blood samples were collected at the tip of the tail before, and 15, 30, 60, 90 and 120 min after administration and glucose concentrations were determined. Moreover, around 20 μL of blood were collected before and 30 min after the injection of glucose and were immediately centrifugated at 4 °C. Plasma samples were stored at −20 °C for the measurement of insulin concentrations.

#### 2.3.6 Insulin tolerance test

After 6 h fasting with free access to water, intraperitoneal insulin tolerance tests (ipITT) were performed in non-anaesthetized GK rats. Rats received intraperitoneal injection human insulin (Novorapid flexpen 100 UI/mL, Novo Nordisk, Bagsværd, Danemark) at the dose of 0.5 U/kg body weight). Blood glucose concentrations were determined in blood samples collected from the tip of the tail, before and 15, 30, 60, 90, 120 min after the injection of insulin.

After 48 h recovery, animals were killed and pancreatic and hepatic tissues were recovered for further analyses.

#### 2.3.7 Histomorphometric analyses

After sacrifice, pancreases were collected and used for histomorphometric studies and the measurement of insulin content. For histological studies, pancreatic tissues were fixed in aqueous Bouin's solution (Sigma Aldrich, Saint Quentin Fallavier, ref. HT10132) and embedded in paraffin (Labonord, Templemars, France). Each paraffin block was serially sectioned (5 μm) throughout its full-length. Pancreatic sections were sampled in a manner to represent the whole pancreatic tissue, and selected sections were mounted on slides and stored at room temperature for future staining. At least 5 homogeneously distributed sections were analyzed for each pancreas.

##### 2.3.7.1 Insulin and glucagon staining

Rehydrated pancreatic sections were blocked for 1 h with 10% normal goat serum in Tris buffer saline and probed for insulin using primary mouse anti-insulin antibody (Santa Cruz Biotechnology, Heidelberg, Germany, ref. sc-8033) or mouse anti-glucagon antibody (Santa Cruz Biotechnology, Heidelberg, Germany, ref. sc-57171) for 1 h. Sections were then incubated with an HRP-conjugated anti-mouse secondary antibody (Jackson Laboratory, Ely, UK). The area of β and α cells were measured using an Olympus BX60 microscope equipped with Histolab 10.5.1 computer-assisted image analysis system software (Microvision Instrument, Evry, France). The relative β or α cell areas in each stained section were calculated as the ratio of insulin or glucagon positive areas over the total tissue area.

##### 2.3.7.2 In vivo β cell proliferation assay

Rehydrated pancreatic sections were blocked for 1 h with 10% normal goat serum in Tris buffer saline and then incubated with mouse anti-BrdU antibody (Cytivia, Dutscher, Bernolshein, France ref. RPN202) for 1 h sections were then incubated with DyLigh 549-conjugated anti-mouse secondary antibody (VectorLabs, Eurobio, Les Ulis, France ref. DI-2549). Sections were then probed for insulin for 1 h (Santa Cruz Biotechnology, Heidelberg, Germany, ref. sc-8033) and then incubated with fluorescent green anti-mouse secondary antibody (Sera Care, USA, ref. 5230-0391). To estimate the β cell proliferation rate, cells double-stained for insulin and BrdU, and those stained for insulin were counted using an Olympus BX40 microscope. Results were expressed as the percentage of double BrdU/insulin-positive cells, over total insulin-positive cells.

#### 2.3.8 Insulin assay

Pancreases were homogenized in an acid-alcohol solution (75% ethanol, 1.5% HCl 32.5 M and 23.5% distilled water) and incubated at 4 °C overnight. Lysates were centrifugated and the supernatants were collected and stored at -20 °C. Pancreatic samples, samples from *ex vivo* insulin secretion studies, and plasma samples recovered at times 0 and 30 min during ipGTTs were assayed for insulin by enzyme linked immunosorbent assay ELISA (Alpco, Eurobio, Les Ulis, France, ref. 80-INSRT-E01) according to the manufacturer's instructions.

#### 2.3.9 Real-time PCR

Total RNAs were extracted from rat liver tissues using the RNeasy mini kit (Qiagen, Courtaboeuf, France, ref. 74104). cDNA of each RNA sample was synthesized with Moloney murine leukemia virus (M-MLV) reverse transcriptase kit (Invitrogen, Illkirch, France, ref. 28025013). Real-time quantitative PCR amplification reactions were carried out in a LightCycler 480 detection system (Roche, Boulogne-Billancourt, France) using the LightCycler 480 SYBR Green 1 Master kit (Roche, Boulogne-Billancourt, France, ref. 04887352001). Primer sets were designed using OLIGO7 and are presented in Table 1. All reactions were run in duplicates. Cyclophilin A was chosen as housekeeping gene, as described in [26,27]. The PCR program was set as follows: 95 °C for 10 min, followed by 40 cycles at 95 °C for 10 s, 60 °C for 10 s and 72 °C for 10 s.

**Table 1:** List of primers.

Gene	Forward	Reverse
***Acc-1***	5’-CCGCCTCTTCCTGACAAAC-3’	5’-CTTGATGAGCGACTGCCGAAA-3’
***Cyclophilin A***	5’-AACCCACCGTGTTCTTC-3’	5’- TGCCTTCTTTCACCTTCCC-3’
***Fasn***	5’-TGTGGCCTTCTCCTCTGTAAGCTG-3’	5’-CGCTTCCAAGATAATGCCCACGTC-3’
***G6Pase***	5’-GTGCCCAGGAAGCCCCACCCATT-3’	5’-AACCCAGCTCCCCATCCCAC-3’
***ChREBP***	5’-CCGGATTCGGACTCGGATAC-3’	5’-CCGCTGTGAATGACCTGTGA-3’
***Glucokinase***	5’-TGGATGAAAGCTCAGCGAAC-3’	5’-TTAAGCAGCACAAGTCGTACCAG-3’
***Glut2***	5’-GAGGCGAGGTTACCATTTCCGAT-3’	5’-AGCGATTGAGTTCAGGTCCTTGT-3’
***Hsl***	5’-CACCCCATTAGCCTTCCCTGTACC-3’	5’-TTTCCCCGCCCCAGGTCCATT-3’
***Lpl***	5’-ACAAGTTTTAGAGCAGGACCAT-3’	5’-AAAACAAAAGAGCGGCAACGA-3’
***Pepck***	5’-ATCCTGGGCATAACTAACCCCGAA-3’	5’-CAGGCAATGTCATCACCCACAC-3’
***LXRα***	5’-ACGAATGACTGTTTCTCCGT-3’	5’-AGCAGCCACAAAACACCCAA-3’
***Srebp1c***	5’-CCTTGCACTTCTTGACACGTT-3’	5’-CCCAGTCCCCATCCACGAA-3’

#### 2.3.10 Data presentation and statistical analyses

Results are expressed as means ± S.E.M. For each data set, the outliers test was performed using the ROUT method (Q = 1%). The statistical significance between means was assessed by Student's *t*-test when two groups were compared. The nonparametric multiple Mann–Whitney test with Holm–Sidak correction was used when data did not meet normal distribution. Ordinary one-way ANOVA followed by Tukey's tests was used when more than two groups were compared. Two-way ANOVA test followed by Bonferroni correction was used when comparing two parameters. All analyses were performed using the GraphPad Prism 9 (GraphPad Software, San Diego, USA). A *p* value less than 0.05 (*p <* 0.05) was considered statistically significant.

## 3 Results

### 3.1 Effect of Leucettinib-92 on pancreatic β cell proliferation

**Figure S1:**
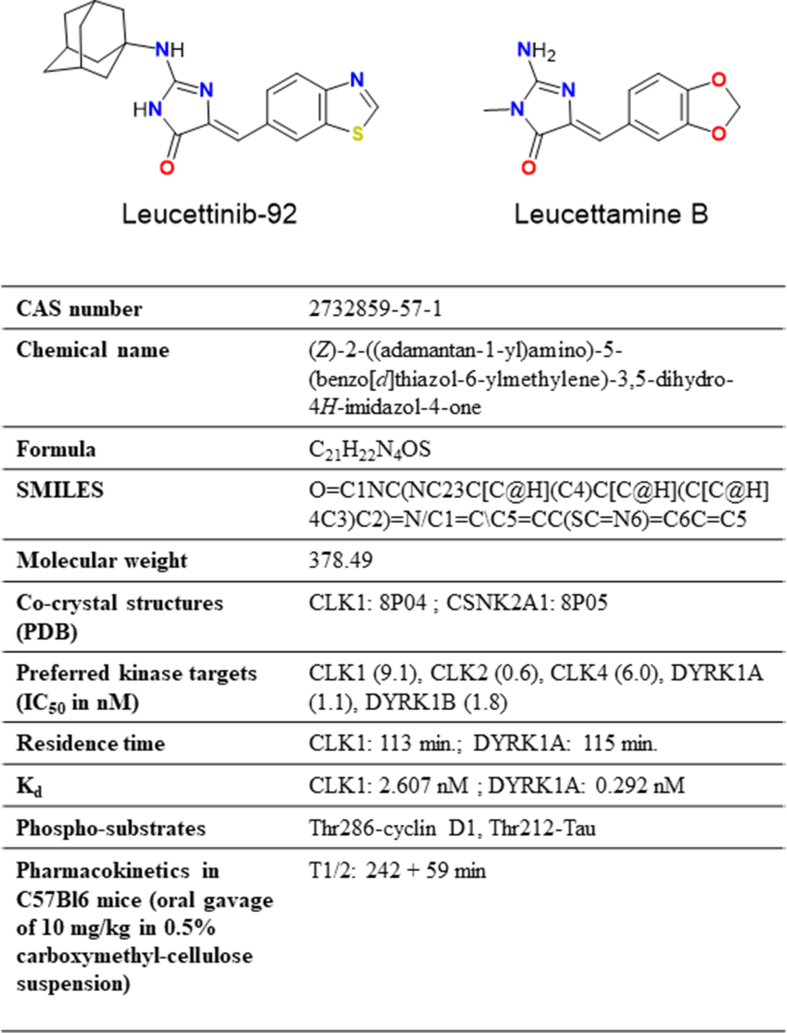
Chemical structure of Leucettinib-92 and the original natural product, Leucettamine B, from which it was inspired. Main physico-chemical and biochemical properties are shown. For more information on LCTB-92 selectivity see ref. [21]. For comparative potency with 56 reported DYRK1A inhibitors, see ref. [22].

In this study we used a DYRK1A inhibitor that belongs to the 2-aminoimidazolin-4-ones family frequently found in marine invertebrates, especially in marine calcareous sponges [21,28]. Inspired by Leucettamine B, a natural product initially identified from the marine sponge *Leucetta microraphis*, and guided by inhibition of a recombinant DYRK1A catalytic activity, a first chemical class, Leucettines, was synthesized [29,30], followed by a second generation of inhibitors, Leucettinibs [21]. Leucettinib-92 was selected as a pharmacological tool for this study. The structure and major properties of Leucettinib-92 (LCTB-92) are shown in Figure S1.

**Figure S2:**
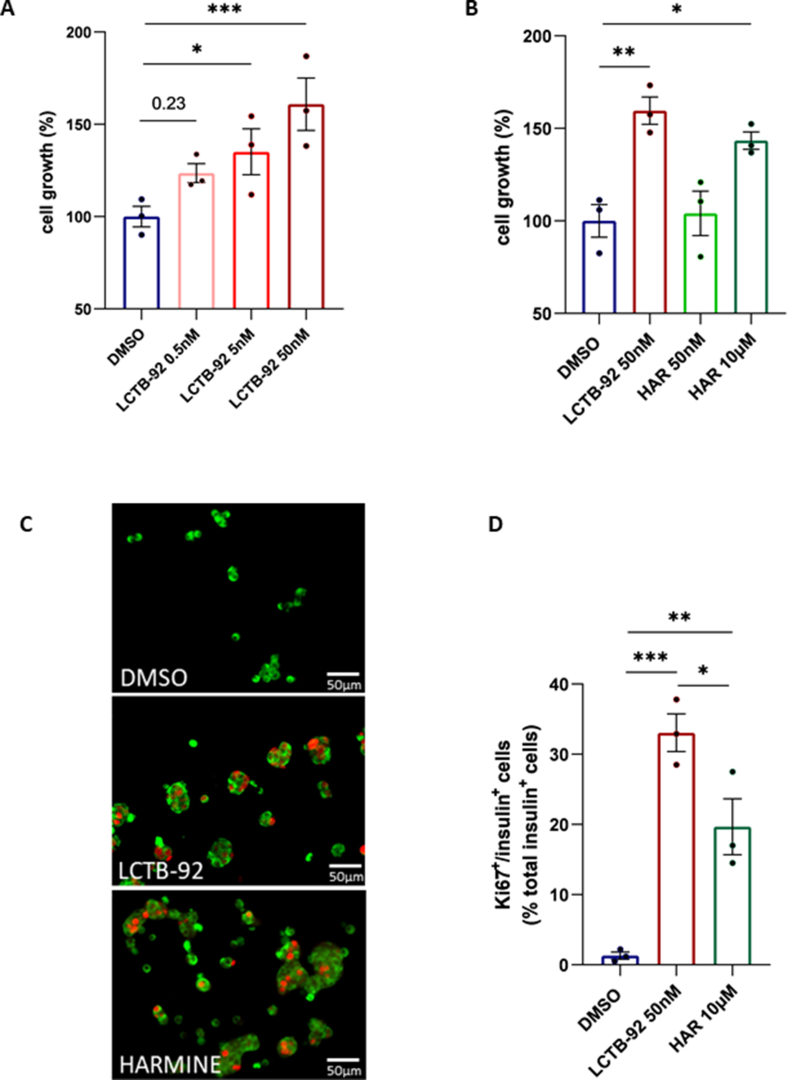
Effect of Leucettinib-92 (LCTB-92) and harmine on β cell proliferation in vitro and *ex vivo*. (A) INS-1 832/13 cells were treated for 48 h with increasing concentrations of LCTB-92 (0.5, 5, and 50 nM). Cell growth was assessed and expressed as a percentage relative to the DMSO-treated control (n = 3). (B) INS-1 832/13 cell proliferation was further evaluated after 48 h of treatment with LCTB-92 (50 nM), harmine (50 nM), or harmine (10 μM). (C) Pancreatic islets isolated from adult Wistar rats were treated *ex vivo* for 72 h with LCTB-92 (50 nM), harmine (10 μM), or DMSO (vehicle control), dissociated by trypsin, and stained by immunofluorescence for insulin (green) and Ki67 (red) (representative image, n = 3). (D) The percentage of insulin/Ki67 double-positive cells was quantified to assess β cell proliferation. Data are presented as means ± S.E.M. n represents the number of independent experiments. Statistical significance was assessed using the non-parametric Mann–Whitney test. ∗p < 0.05; ∗∗p < 0.01; ∗∗∗p < 0.001.

Numerous pharmacological inhibitors of DYRK1A belonging to various chemical classes have been shown to induce the proliferation of β cells in vitro [[14], [15], [16]]. We first wanted to verify that Leucettinib-92 shared the same property. INS-1 823/13 rat insulinoma β cells were exposed to a range of Leucettinib-92 concentrations, in order to identify the suitable dose to be used in primary β cells. The assessment of cell growth in INS-1 823/13 β cells showed that concentrations as low as 5 nM and 50 nM exert significant growth-stimulating effects in this β cell line (Figure S2A).

Next, we performed additional experiments to benchmark the effects of Leucettinib-92 against harmine, one of the most commonly used DYRK1A inhibitors. We show that the effects of Leucettinib-92 on cell growth are markedly stronger than harmine, since harmine at the same concentration as Leucettinib-92 (50 nM) failed to induce cell growth in INS-1 823/13 cells. It was only at the concentration of 10 μM that harmine induced effects comparable to Leucettinib-92 at 50 nM (Figure S2B).

**Figure 1:**
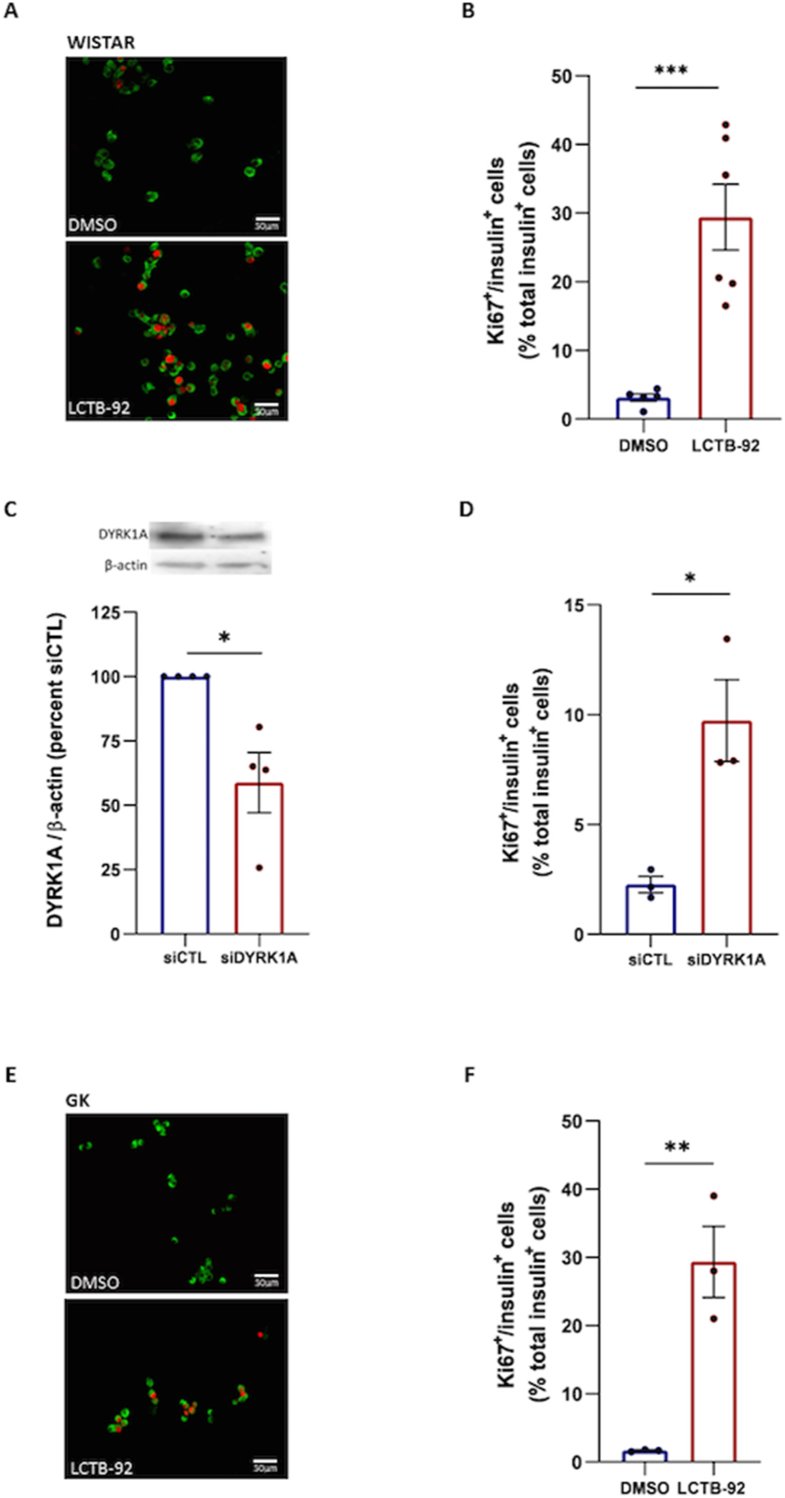
Effects of the DYRK1A inhibitor Leucettinib-92 (LCTB-92) in cultured pancreatic β-cell proliferation. (A) Pancreatic islets isolated from Wistar rats treated for 72 h with the pharmacological DYRK1A inhibitor Leucettinib-92 (50 nM) or DMSO (vehicle) were dissociated with trypsin and immunostained for insulin (green) and Ki67 (red) (representative image, n = 6). (B) The percentage of double-positive insulin/Ki67 cells was quantified. (C) Cells obtained after dissociation of Wistar rat islets were transfected with siDYRK1A or siCTL and cultured for 48 h. DYRK1A protein expression was assessed by western blotting (n = 3). (D) Transfected cells (siDYRK1A or siCTL) were immunostained for insulin and Ki67, and the percentage of double-positive insulin/Ki67 cells was assessed (n = 3). (E) The same experiments as in (A) were performed with islets isolated from diabetic GK rats. (F) Immunostained cells (insulin in green, Ki67 in red) were quantified. Results are presented as the percentage of double-positive insulin/Ki67 cells over total insulin-positive cells (n = 3). Data are presented as means ± S.E.M. n represents the number of independent experiments. Statistical significance was assessed using the non-parametric Mann–Whitney test. ∗p < 0.05; ∗∗p < 0.01; ∗∗∗p < 0.001.

Dispersed islets isolated from pancreases of healthy Wistar rats were next exposed to 50 nM Leucettinib-92. A nine-fold increase in β cell proliferation was observed after 72 h of Leucettinib-92 treatment, compared with the proliferation rate of β cells in vehicle (DMSO)-treated islets (Figure 1A, B).

Next, we compared the proliferative effects of Leucettinib-92 (50 nM) to harmine (10 μM), and showed that the percentage of Ki67^+^ β cells was significantly higher in cells treated with Leucettinib-92 compared to cells treated with harmine (Figure S2C; S2D), indicating that Leucettinib-92 is a more potent inducer of β cell proliferation than harmine.

The implication of DYRK1A in these observations was confirmed using siRNAs targeting DYRK1A (siDYRK1A). Transfection of islets from Wistar rats with siDYRK1A resulted in a significant reduction in DYRK1A protein levels (Figure 1C). Ki67 quantification revealed that siRNA-mediated knockdown of DYRK1A resulted in a 4-fold increase in β cell proliferation compared with the proliferation rate found in islets treated with control siRNA (siCTL) (Figure 1D).

To evaluate the effect of DYRK1A inhibition in diabetic β cells, dispersed islets isolated from pancreases of 3-month-old diabetic GK rats were treated with Leucettinib-92 for 72 h. A significant increase in β cell proliferation was observed in cells treated with Leucettinib-92 (Figure 1E, F).

These findings identify Leucettinib-92 as a potent inducer of pancreatic β cell proliferation and confirm the involvement of DYRK1A in this process, as seen with other DYRK1A inhibitors in β cells of different origins [7,17]. Furthermore, Leucettinib-92 successfully stimulated the proliferation of β cells obtained from diabetic GK rats, encouraging the investigation of a potential therapeutic benefit of Leucettinib-92 in diabetic settings.

### 3.2 Effect of Leucettinib-92 on insulin secretion by β cells

**Figure 2:**
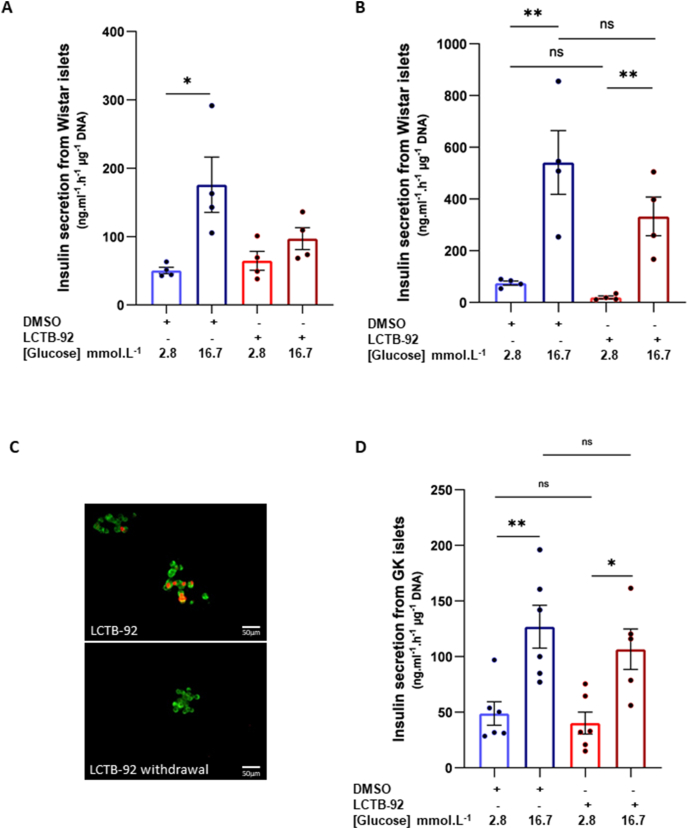
Effect of Leucettinib-92 (LCTB-92) on insulin secretion by isolated islets. (A) Insulin secretion measurement in Wistar rat pancreatic islets after 72 h incubation with or without LCTB-92 (n = 4). Insulin secretion was assessed in media samples collected from islets incubated with either 2.8 mM or 16.7 mM glucose. Insulin levels in the media samples were measured by ELISA and expressed as ng/ml/hour/μg DNA per islet. (B) Insulin secretion measurement in Wistar rat pancreatic islets following 72 h incubation with or without LCTB-92, followed by 96 h of culture without treatment (n = 4). Insulin secretion was assessed as described in (A). (C) Immunostaining of insulin (green) and Ki67 (red) in islets treated as described in (B), followed by trypsin dissociation. (D) Insulin secretion measurement in GK rat pancreatic islets after 72 h incubation with or without LCTB-92, followed by 96 h culture without treatment (n = 5). Data are presented as means ± S.E.M. n represents the number of independent experiments. Statistical significance was assessed using the non-parametric Mann–Whitney test. ∗p < 0.05; ∗∗∗p < 0.001.

Glucose-induced insulin secretion assays were next performed to assess the functional integrity of isolated islets exposed to Leucettinib-92 for 72 h. During the active proliferative phase induced by Leucettinib-92, islets isolated from Wistar rats showed decreased glucose-stimulated insulin secretion compared with DMSO-treated islets which had low proliferative activity (Figure 2A). Active proliferation is thus likely to inhibit glucose-induced insulin secretion.

A wash-out study was then carried out to evaluate the reversibility of this inhibition. Islets were cultured with Leucettinib-92 for 72 h and subsequently deprived of the inhibitor for the following 4 days. At that point, glucose-induced insulin secretion abilities were fully restored, reaching levels comparable to that of DMSO-treated islets (Figure 2B). Notably, removal of Leucettinib-92 from the culture medium halted β cell proliferation (Figure 2C).

Similar experiments conducted in islets isolated from diabetic GK rats demonstrated that after the induction of proliferation, β cells cultured without Leucettinib-92 recovered their initial glucose-triggered insulin secretion capacity (Figure 2D). As expected, the insulin secretory capacity of islets from GK rats remained lower than that of Wistar rat islets.

These results show that the normal insulin secretory function of β cells is restored in post-mitotic β cells, after a transient impairment occurring during the proliferative phase.

### 3.3 Effect of short-term Leucettinib-92 treatment in 5-week-old GK rats

#### 3.3.1 β cell mass and proliferation following 5-day Leucettinib-92 treatment

A specific feature of GK rats is the presence of a prediabetic phase during the first 4 postnatal weeks, followed by the development of diabetes within one to two weeks after weaning [31].

Our first set of *in vivo* experiments was conducted in 5-week-old juvenile GK rats that had not yet developed overt hyperglycemia, which we considered as > 200 mg/dL. Rats were treated daily for 5 days by intraperitoneal injection of Leucettinib-92 (0.5 mg/kg body weight) or vehicle (DMSO).

**Figure S3:**
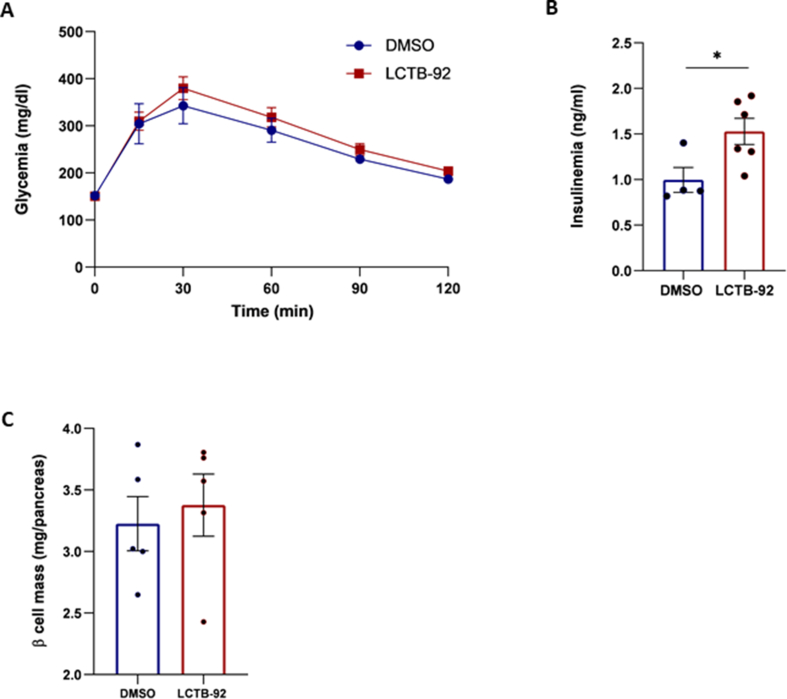
Effect of short-term Leucettinib-92 (LCTB-92) treatment on glucose tolerance and insulinemia in 5-week-old GK rats. Five-week-old GK rats were treated for 5 days with LCTB-92 (0.5 mg/kg body weight, intraperitoneally) (n = 6) or with DMSO (n = 4). (A) An intraperitoneal glucose tolerance test (ipGTT) was performed at the end of the treatment. Blood glucose levels were measured at baseline and at 15, 30, 60, 90, and 120 min following intraperitoneal injection of glucose (1 g/kg body weight). (B) Basal insulinemia was measured at 9:00 a.m. In parallel, five-week-old Wistar rats were treated for 5 days with LCTB-92 (0.5 mg/kg body weight, intraperitoneally) (n = 5) or with DMSO (n = 5). (C) At the end of the treatment, pancreata were collected, immunostained for insulin, and β-cell mass was quantified. Data are presented as means ± SEM. Statistical significance was assessed using the non-parametric Mann–Whitney test. ∗p < 0.05.

In Leucettinib-92 and DMSO-treated animals, we performed an ipGTT and found no difference in glucose tolerance between the two groups (Figure S3A). Interestingly the random-fed plasma insulin levels at the end of treatment was higher in LCTB-92 rats compared to DMSO-treated animals (Figure S3B).

**Figure 3:**
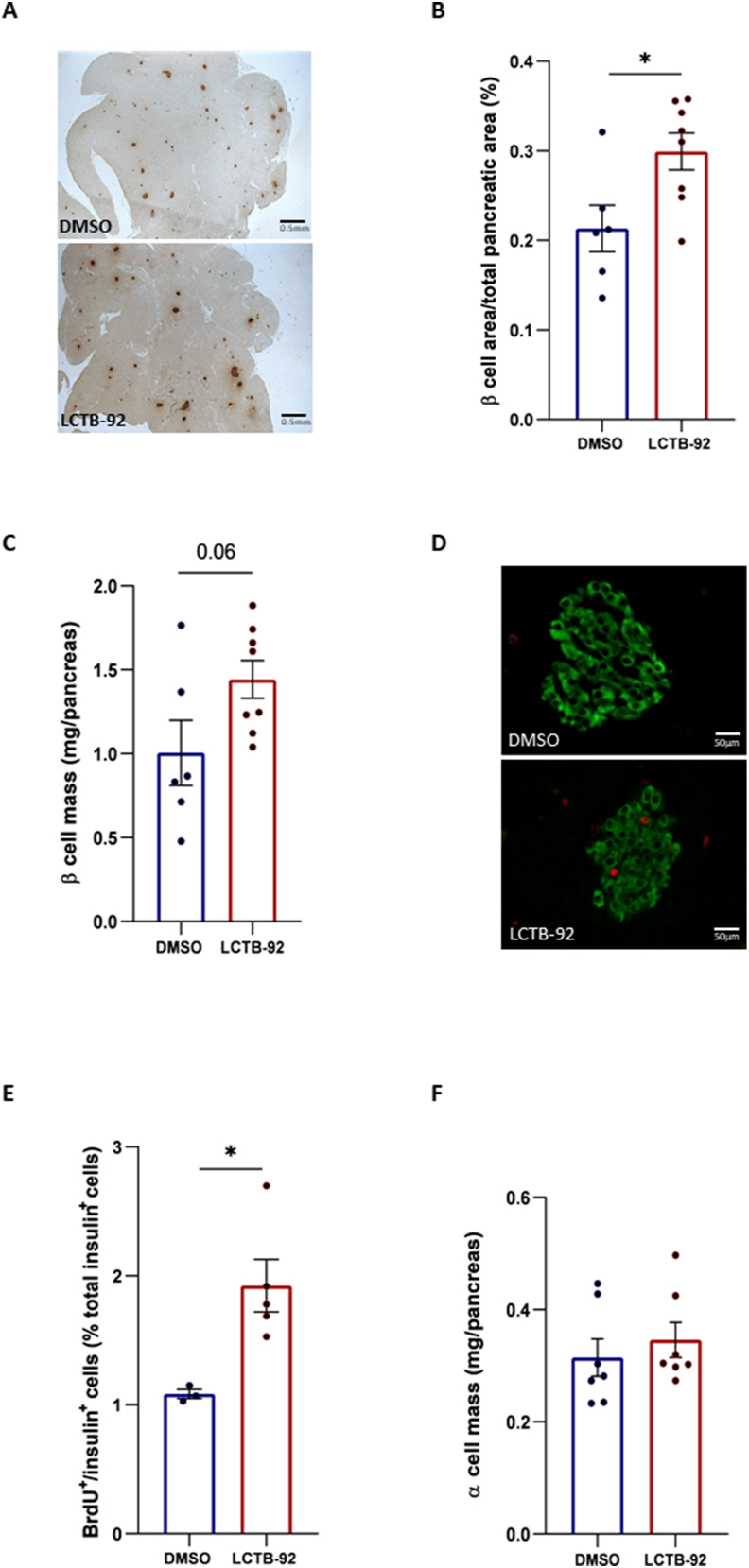
Effect of short-term Leucettinib-92 (LCTB-92) treatment on pancreatic β cell proliferation and mass in 5-week-old GK rats. 5-week-old GK rats were treated for 5 days with LCTB-92 (0.5 mg/kg body weight, intraperitoneal) (n = 8) or DMSO (n = 6). At the end of the treatment, rats were sacrificed, and pancreases were immunostained for insulin (A). (B) The relative β cell area was quantified. (C) β cell mass and α cell mass (F) were calculated by multiplying respectively the relative β and α cell areas to the pancreatic tissue weight. (D) Pancreatic sections were co-immunostained for insulin (green) and BrdU (red), and the percentage of double-positive cells was assessed (E) (n = 4). Data are presented as means ± S.E.M. Statistical significance was assessed using the non-parametric Mann–Whitney test. ∗p < 0.05.

Following the treatment, pancreases from animals in each group were harvested for analysis. Histomorphometric analyses of pancreatic sections revealed a 45% increase in the β cell area in Leucettinib-92-treated GK rats compared with vehicle-treated GK rats (Figure 3A, B). A similar trend (p = 0.06) was observed for the pancreatic β cell mass (Figure 3C), which was increased by about 42% in the Leucettinib-92-treated rats compared to DMSO-treated rats. Additionally, BrdU staining quantification demonstrated a significant increase in β cell proliferation in Leucettinib-92-treated rats (Figure 3D, E). These findings indicate that Leucettinib-92 enhances β cell mass *in vivo* under diabetic conditions, by promoting β cell proliferation. In addition, the evaluation of the α cell mass revealed no significant difference between Leucettinib-92 and DMSO -treated groups (Figure 3F), suggesting a β cell-specific proliferative effect of DYRK1A inhibition.

Finally, the quantification of the β cell surface area and the calculation of the β cell mass in non-diabetic 5-week-old Wistar rats showed no significant difference between Leucettinib-92 and DMSO -treated rats (Figure S3C), suggesting that, as previously reported [4,32], in the absence of β cell deficiency, cell homeostasis is maintained within the tissues, preventing the generation of unwanted new cells.

#### 3.3.2 Glycemia, β cell mass and insulin content following treatment discontinuation

**Figure 4:**
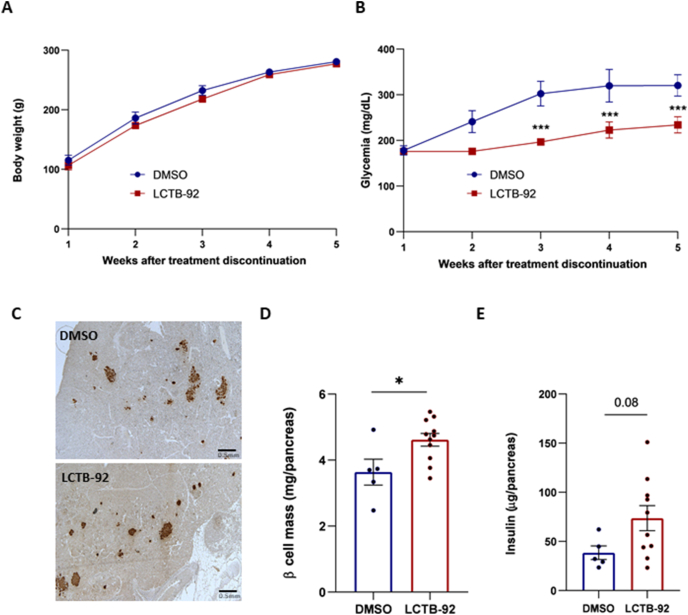
Five-week post-treatment study of the effect of Leucettinib-92 (LCTB-92) on glycemic evolution, pancreatic β cell mass, and insulin content in GK rats. Pre-diabetic 5-week-old GK rats were treated for 5 days with LCTB-92 (0.5 mg/kg body weight, intraperitoneal) (n = 10) or DMSO (n = 5). After treatment, (A) body weight and (B) blood glucose levels were monitored for 5 weeks. (C) Pancreatic sections were immunostained for insulin. (D) β cell was calculated as described in Figure 3, and (E) pancreatic insulin content was measured in pancreatic homogenates by ELISA. Two-way ANOVA followed by Bonferroni's multiple comparisons test was used for panels A and B. Statistical significance was determined using the non-parametric Mann–Whitney test was used for panels D and E. ∗p < 0.05; ∗∗∗p < 0.001.

In another group of rats treated daily with Leucettinib-92 (0.5 mg/kg body weight, i.p.) for 5 days, the treatment was discontinued and rats were monitored during the 5 following weeks. The growth pattern, reflected by the body weight values, was comparable between the control and Leucettinib-92-treated groups, confirming the lack of toxicity and good tolerance of the drug (Figure 4A). In contrast, the glycemic profiles differed significantly between the Leucettinib-92 and vehicle -treated groups. While blood glucose levels increased gradually in control GK rats to reach the glycemic value of 320.4 +/− 52.3 mg/dL at the end of the follow-up period, the glycemia of Leucettinib-92-treated rats was 234.0 +/− 62.3 mg/dL at that time point (Figure 4B). Thus, Leucettinib-92 treatment resulted in 27% reduction of glycemia compared with control GK rats, 5 weeks after treatment discontinuation.

Histological analysis of the pancreas at the end of the follow-up period revealed that Leucettinib-92-treated rats displayed an increased β cell mass compared with untreated rats (Figure 4C, D), similar to the effect observed immediately at the end of treatment. Pancreatic insulin content also showed a marked trend toward higher levels in Leucettinib-92-treated GK rats compared to untreated animals (Figure 4E).

These results suggest that a short-term (5 days) Leucettinib-92 treatment in pre-diabetic GK rats is sufficient to induce a sustained increase in β cell mass and long-term (5 weeks) mitigation of hyperglycemia.

### 3.4 Effect of long-term Leucettinib-92 treatment in adult diabetic GK rats

**Figure S4:**
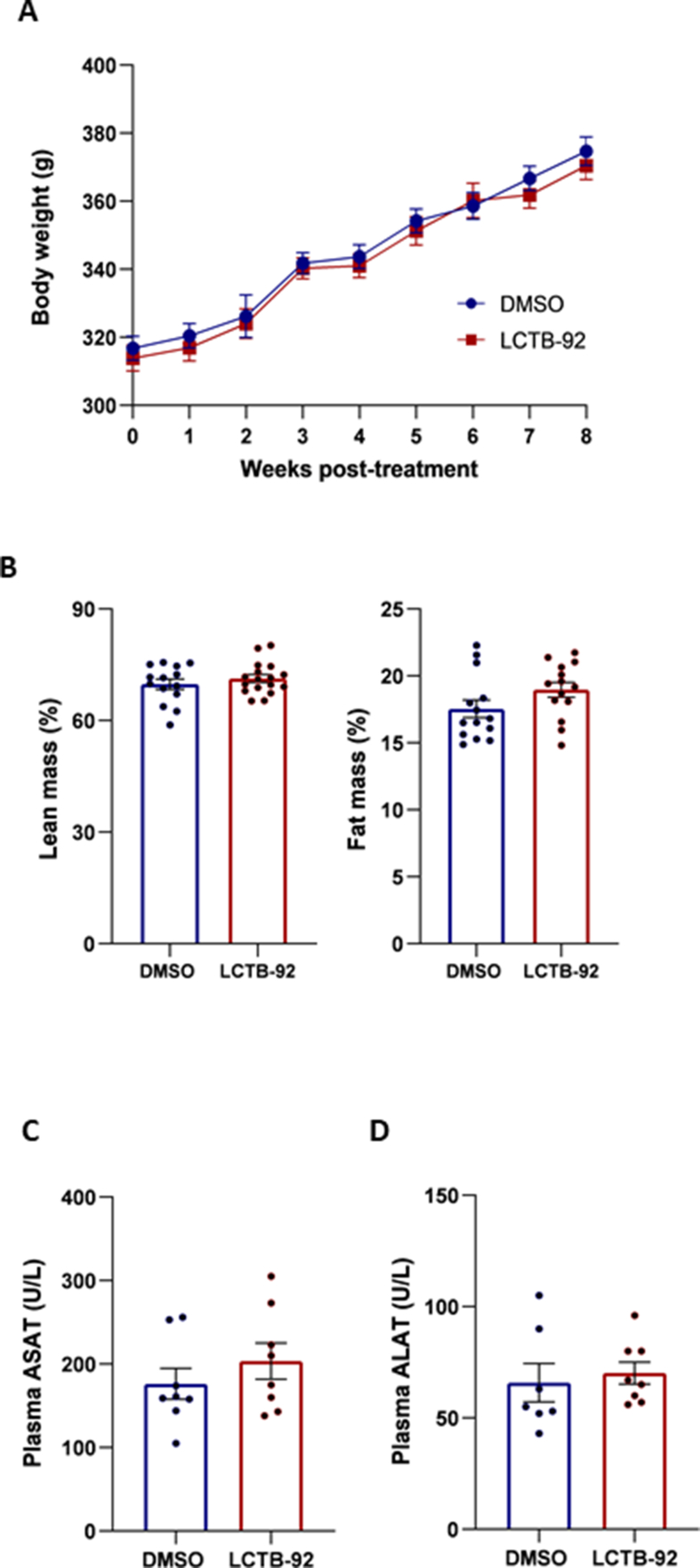
Effect of long-term Leucettinib-92 (LCTB-92) treatment on diabetic GK rats. Three-month-old GK rats were treated daily with LCTB-92 (0.05 mg/kg body weight, intraperitoneal) (n = 16) or DMSO (n = 21) for 8 weeks. (A) Body weight was recorded weekly. (B) Body composition was assessed by the evaluation of fat mass, and lean mass using EchoMRI at the end of treatment period (LCTB-92 n = 14; DMSO n = 15). Plasma levels of aspartate aminotransferase (ASAT) (C) and alanine aminotransferase (ALAT) (D) were measured in GK rats after daily treatment with LCTB-92 (0.05 mg/kg body weight, intraperitoneal) (n = 8) or DMSO (n = 7) for 8 weeks.

To evaluate whether β cell regeneration can be induced in the pancreas of adult animals with established diabetes, 3-month-old diabetic GK rats were treated daily for 8 consecutive weeks with Leucettinib-92 (0.05 mg/kg body weight, i.p.). Body weight and blood glucose levels were monitored weekly. Long-term treatment with Leucettinib-92 did not alter the body weight gain in comparison with DMSO-treated rats (Figure S4A), again demonstrating the drug's innocuity, even over extended periods. In addition, no macroscopic physical or behavioral alteration were observed in the Leucettinib-92 group throughout the course of the treatment. The assessment of body composition showed no significant difference in the percentage of fat mass and lean mass between the two groups (Figure S4B). Moreover, the measurement of aspartate and alanine transaminases (ASAT and ALAT) in plasma samples collected at the end of the 8-week treatment period showed no difference between the vehicle and Leucettinib-92 -treated groups, suggesting the absence of drug's hepatotoxicity, even during a long-term treatment (Figure S4C and S4D). Furthermore, since a potential tumorigenic effect of DYRK1A inhibition has been suggested (for review see [33]), in our present study, we carefully examined anatomical aspects of most abdominal organs and also the brain in euthanized animals, and we did not observe any abnormal tissue development, at least at macroscopic level, in rats treated with Leucettinib-92 for 8 consecutive weeks.

**Figure 5:**
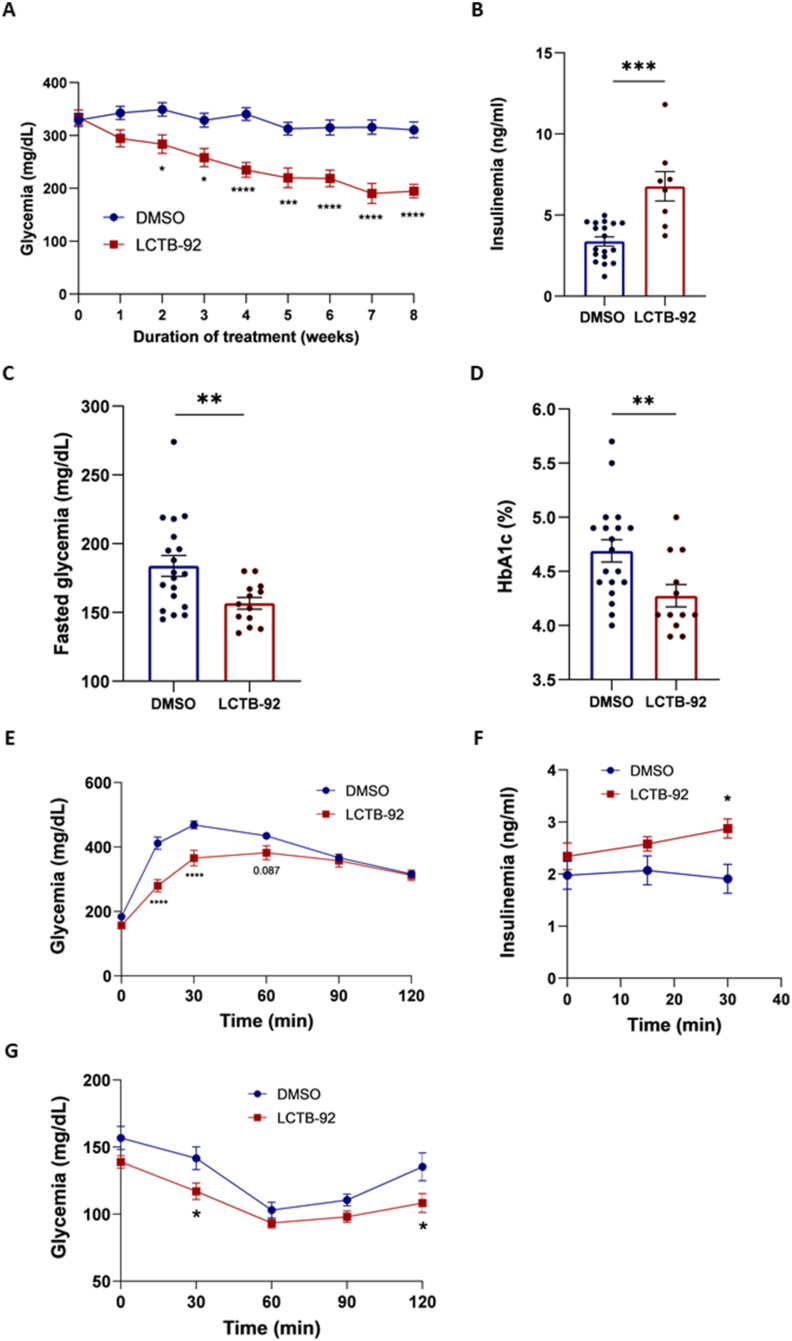
Effect of long-term Leucettinib-92 (LCTB-92) treatment on diabetic GK rats. Three-month-old GK rats were treated daily with LCTB-92 (0.05 mg/kg body weight, intraperitoneal) (n = 16) or DMSO (n = 21) for 8 weeks. (A) Non-fasted blood glucose levels were measured weekly throughout the 8-week treatment period. (B) At the eighth week of treatment, basal insulinemia measured at 9:00 a.m., (C) blood glucose was measured after 6 h of fasting, and (D) glycated hemoglobin (HbA1c) levels were quantified. (E) During the final week of treatment, an intraperitoneal glucose tolerance test (ipGTT) was performed. Blood glucose levels were measured before and 15, 30, 60, 90, 120 min following the intraperitoneal injection of 1 g glucose/kg body weight. (F) Plasma insulin levels were measured in samples collected at t = 0, t = 15 min and t = 30 min after intraperitoneal injection of glucose during ipGTT. (G) An insulin tolerance test (ITT) was performed after 6 h of fasting, with blood glucose measured before and 30, 60, 90, and 120 min following the intraperitoneal injection of 0.5 U insulin/kg body weight. Results are shown as means ± S.E.M. n represents the number of animals. Statistical significance was determined using the non-parametric Mann–Whitney test was used for panels B, C, and D. Two-way ANOVA followed by Bonferroni's multiple comparisons test was used for panels A, E, and G. ∗p < 0.05; ∗∗p < 0.01; ∗∗∗p < 0.001.

Random-fed GK rats treated with Leucettinib-92 displayed significant reduction of blood glucose levels. In contrast, glycemia measured in control rats remained essentially the same as the initial glycemia (Figure 5A). At the end of the 8-week treatment period, glycemia in Leucettinib-92-treated animals was 204.7 +/− 14 mg/dL, which represented a 37.3 % reduction compared to the glycemia of vehicle-treated animals (317 +/− 23 mg/dL) (Figure 5A). Random-fed insulinemia in Leucettinib-92 treated rats was significantly higher than that in DMSO-treated rats (Figure 5B). This could partly explain the reduced basal hyperglycemia seen in LCTB-treated rats in Figure 5A. Interestingly, glycemia measured after 6 h of fasting was significantly lower in the Leucettinib-92-treated group (Figure 5C) compared to the control group. Finally, the rate of glycated hemoglobin (HbA1c), a relevant marker for glycemic control assessment, was significantly reduced in the Leucettinib-92-treated group (Figure 5D).

#### 3.4.1 Metabolic functions

Metabolic tests were next performed in animals to investigate the consequences of the Leucettinib-92-triggered improvement of random-fed and fasted glycemia. Intraperitoneal glucose tolerance tests (ipGTT) were run in both Leucettinib-92 and vehicle -treated GK rats. Results revealed improved glucose tolerance (Figure 5E) and enhanced glucose-induced insulin secretion in Leucettinib-92-treated GK rats (Figure 5F). However, no marked amelioration of insulin action was found in the Leucettinib-92-treated group compared to the DMSO-treated group (Figure 5G).

These results demonstrate that chronic Leucettinib-92 treatment in adult diabetic GK rats significantly improves metabolic outcomes, especially through its effects on glucose tolerance and insulin secretion.

#### 3.4.2 β cell mass and pancreatic insulin content

**Figure 6:**
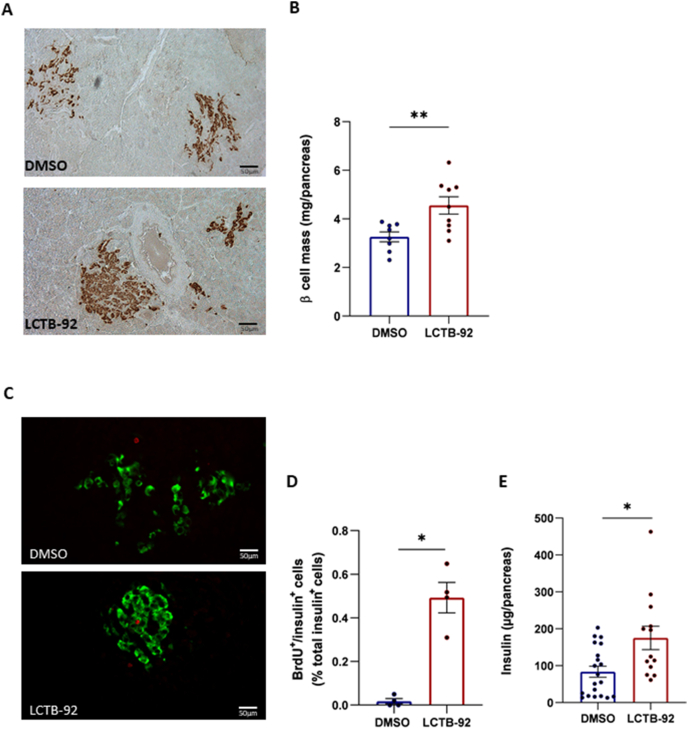
Effect of long-term Leucettinib-92 (LCTB-92) treatment on diabetic GK rats. (A) Representative pancreatic sections were immunostained for insulin (brown) to visualize islet morphology and insulin-producing β cells. (B) β cell mass was measured by morphometric analysis of insulin-stained sections. (C) β cell proliferation was evaluated by double immunofluorescence staining for insulin (green) and BrdU (red). (D) The percentage of insulin-positive β cells incorporating BrdU was quantified to assess the proportion of proliferating β cells. And (E) pancreatic insulin content were quantified. Results are shown as means ± S.E.M. n represents the number of animals. Statistical significance was determined using the non-parametric Mann–Whitney test. ∗p < 0.05; ∗∗p < 0.01.

At the end of the 8-week treatment period, the β cell mass was significantly increased in Leucettinib-92-treated GK rats compared with vehicle-treated control rats (Figure 6A, B). The increase of the β cell mass in Leucettinib-92 rats was associated with increased β cell proliferation assessed by the evaluation of BrdU incorporation in β cells of adult rats treated with Leucettinib-92 (Figure 6C, D). Consistent with this observation, the pancreatic insulin content was higher in the Leucettinib-92-treated GK rats (Figure 6E).

#### 3.4.3 Plasma lipid profile and hepatic regulation of glucose and lipid metabolism

**Figure 7:**
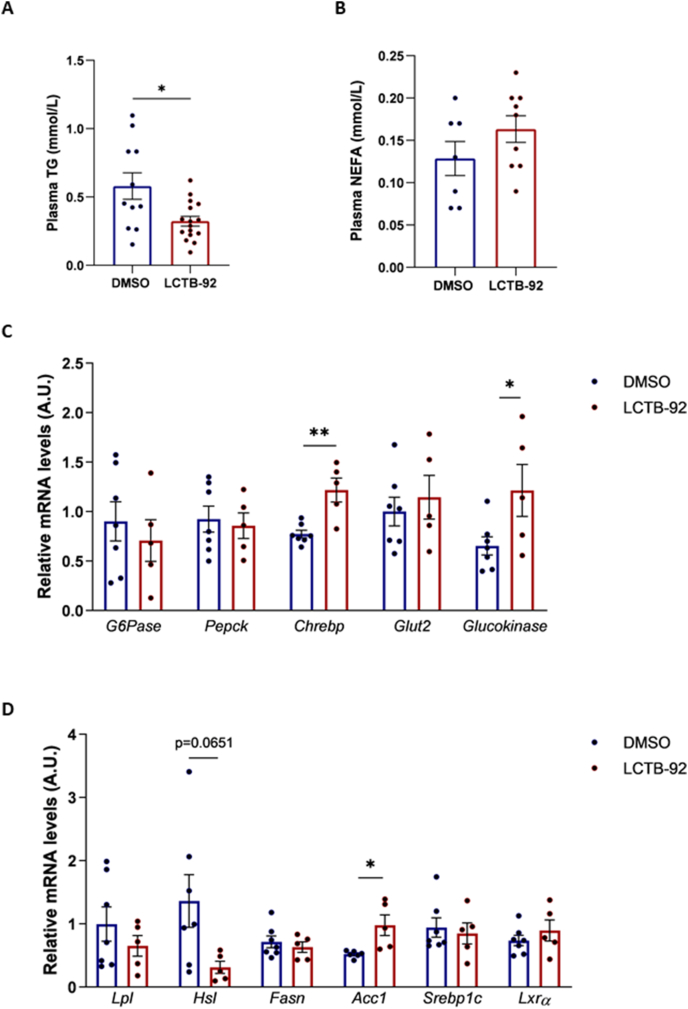
Effects of Leucettinib-92 (LCTB-92) treatment on plasma lipid levels and hepatic gene expression in GK rats. Triacylglycerol (TG) (A) and non-esterified fatty acid (NEFA) (B) levels were measured in the plasma of GK rats following daily treatment with LCTB-92 (0.05 mg/kg body weight, intraperitoneal) or DMSO for 8 weeks (TG: DMSO n = 11; LCTB-92 n = 16. NEFA: DMSO n = 7; LCTB-92 n = 9). Relative mRNA levels of genes involved in glucose metabolism (C) or lipid homeostasis (D) were measured in the liver of GK rats treated for 8 weeks with LCTB-92 (0.05 mg/kg body weight, intraperitoneal) (n = 5) or DMSO (n = 7). Data are presented as means ± S.E.M. n represents the number of animals. Statistical significance was assessed using the non-parametric Mann–Whitney test. ∗p < 0.05; ∗∗p < 0.01.

Concentrations of circulating triglycerides and non-esterified fatty acids (NEFA) were determined at the end of the 8-week treatment period. Leucettinib-92 treatment lowered triglycerides levels (Figure 7A) while it had no significant effect on NEFA levels (Figure 7B). Furthermore, we assessed the expression of major genes involved in hepatic glucose and lipid metabolism. We found that the levels of *glucokinase* and *carbohydrate-responsive* *element-binding protein* (*ChREBP)* mRNAs were increased in liver of Leucettinib-92-treated rats, while the levels of *glucose transporter-2* (*Glut2)*, *glucose-6 phophatase (G6Pase)* and *phosphoenolpyruvate carboxykinase* (*PEPCK)* were similar between the two groups(Figure 7C). Regarding genes involved in lipid metabolism, we found that the expression of *acetyl-CoA* *Carboxylase* (*ACC1*) and *hormone sensitive lipase* (*HSL*) was, respectively, increased and decreased, while mRNA levels of other genes remained unchanged in rats treated with Leucettinib-92 compared to vehicle-treated rats (Figure 7D).

Finally, in order to evaluate whether Leucettinib-92 treatment alters glucose homeostasis in non-diabetic Wistar rats, we treated 3-month-old male Wistar rats with LCTB-92 or DMSO for 6 weeks.

**Figure S5:**
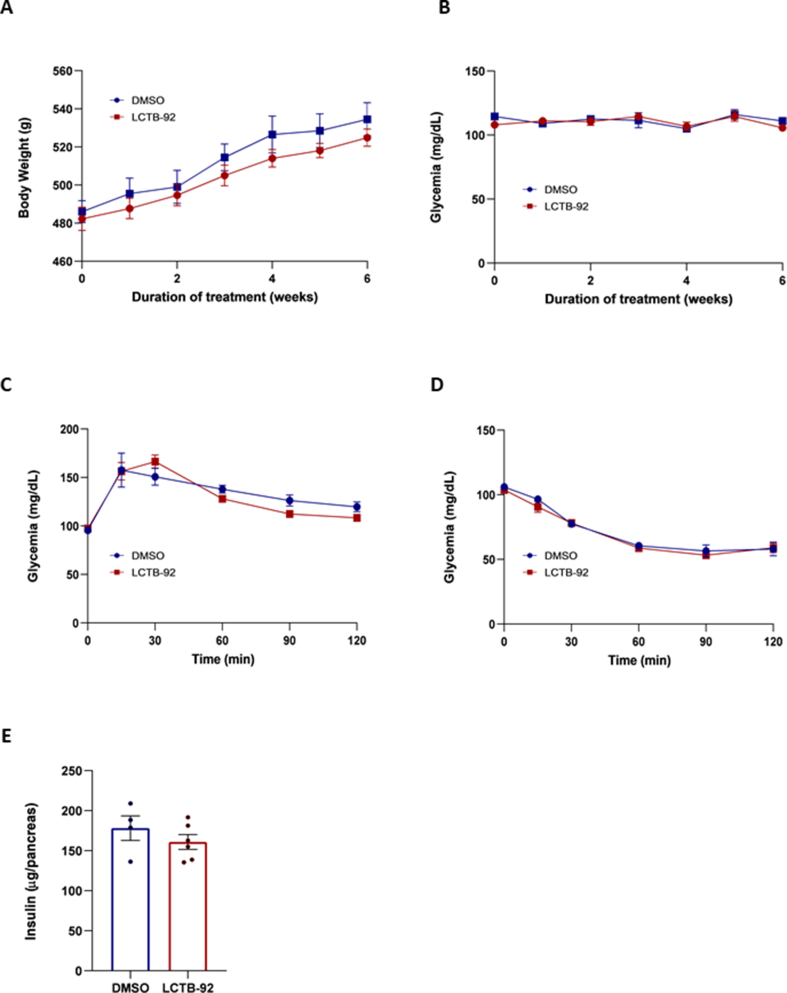
Effect of long-term Leucettinib-92 (LCTB-92) treatment in non-diabetic Wistar rats. Three-month-old Wistar rats were treated daily for 6 weeks with LCTB-92 (0.05 mg/kg body weight, intraperitoneally) (n = 6) or with DMSO (n = 4). (A) Body weight and (B) blood glucose levels were measured weekly throughout the 6-week treatment period. (C) During the final week of treatment, an intraperitoneal glucose tolerance test (ipGTT) was performed. Blood glucose levels were measured at baseline and at 15, 30, 60, 90, and 120 min following intraperitoneal injection of glucose (1 g/kg body weight). (D) An insulin tolerance test (ITT) was conducted after 6 h of fasting, with blood glucose measured at baseline and at 30, 60, 90, and 120 min following intraperitoneal injection of insulin (0.5 U/kg body weight). (E) At the end of the 6-week treatment, pancreatic insulin content was measured by ELISA.

The evaluation of body weight gain showed no difference between the two groups, again reflecting the innocuity of this treatment, even when administered during a long period of time (Figure S5A).

This treatment has no effect on basal glycemia (Figure S5B) and the glycemic profile was similar in LCTB-92 and DMSO groups during glucose tolerance tests (Figure S5C) and insulin tolerance tests (Figure S5D).

Moreover, the pancreatic insulin content was not affected by long-term treatment with Leucettinib-92 (Figure S5E), suggesting that in the context of normal glucose metabolism (non-diabetic rats), DYRK1A inhibition does not affect animal's glycemic status, and thus has no risk of unwanted hypoglycemia.

Interestingly, one should note that the pancreatic insulin content of adult GK rats treated with LCTB-92 (Figure 6E) nears that of non-diabetic Wistar rats (Figure S5E), illustrating the strong positive effect of the inhibitors on the restoration of pancreatic insulin levels.

## 4 Discussion

The total (T1D) or partial (T2D) loss of functional β cells is the root-cause of diabetes pathogenesis. To date, the current medications used to treat diabetic patients provide only a symptomatic relief without addressing the underlying cause of insulin deficiency.

As such, there is a strong unmet medical need for therapeutics that address a major underlying cause, namely the loss of β cells leading to overt hyperglycemia. One approach to achieve the replenishment of the β cell population within the pancreas is based on implanting a novel source or restoring an endogenous source of insulin-producing cells. Islet transplantation is currently used in a limited population of T1D diabetic patients who are under immunosuppressive drugs as a consequence of prior kidney transplantation [34]. However, this therapy is not applicable to T2D diabetes because of both the shortage of islet donors and the need for immunosuppression, which has no medical justification in T2D patients. An alternative strategy for the restoration of β cell mass in patients with T2D is to foster *in vivo* β cell regeneration from patient's endogenous cell sources.

Pharmacological interventions that could stimulate β cell proliferation within the pancreas thus appear as a straightforward promising strategy to address diabetes. However, it should be noted that T2D is a heterogenous disease and in different populations, differing degrees of increased insulin resistance and decreased insulin secretion are involved in the pathogenesis of T2D [1,2,35]. Therefore, T2D patients with severe insulin resistance and moderate insulin deficiency are not good candidates for β cell regenerative therapies.

During the past decade, DYRK1A has been identified as a negative regulator of β cell proliferation. Harmine and its analogues are among the first and most commonly studied DYRK1A inhibitors [7,9,36]. However, their use *in vivo* is associated with undesired secondary targets and side effects [37]. Other compounds with more or less selective DYRK1A inhibitory effects have been used by several research groups [7,9,10,17,22].

Our results validate the proliferation-stimulating effect of a Leucettinib DYRK1A inhibitor on primary islets isolated from healthy Wistar rats. The rate of proliferation achieved by Leucettinib-92 at low concentration (50 nM) was much higher than that reported with other inhibitors at higher (micromolar) concentrations [15,38,39]. Furthermore, 4 days after treatment discontinuation, β cells retained normal glucose-induced secretory function, suggesting that newly generated β cells were fully functional. Most DYRK1A inhibitors also inhibit DYRK1B, as well as some closely related kinases such as CLK1, CLK4 [16,22,40], and, for some of them, other kinases such as GSK3 [17]. Leucettinib-92 inhibits DYRK1A, DYRK1B, DYRK2, DYRK3, and DYRK4 with IC50 values of 1.2, 1.8, 40.2, 19.3, and 117.4 nM, respectively [21]. It inhibits CLK1, CLK2, CLK3, CLK4, and GSK3β with IC50 values of 9.2, 0.6, 811.6, 6.0 nM and 4,158 μM, respectively. Given the high IC50 values of LCTB-92 for CLK3 and GSK3β, the contribution of inhibition of these enzymes to the stimulation of β cell proliferation is unlikely [41]. On the other hand, based on the strong inhibitory effect of LCTB-92, we cannot exclude the contribution of DYRK1B, CLK1, CLK2 and CLK4. However, CLK 1, 2, and 4 should not be involved in cell proliferation, since their silencing, individually or in combination, had no effect on human β cell proliferation [16]. In contrast, the contribution of DYRK1B inhibition by LCTB-92 to β cell proliferation is rather likely, as shown by Ackeifi and colleagues [16]. As a complement to the pharmacological inhibition by Leucettinib-92 which has high selectivity for a few DYRKs and CLKs, we performed genetic manipulation in order to confirm the specific implication of DYRK1A in cell proliferation. We showed that siRNA-mediated knockdown of DYRK1A leads to a significant increase of β cell proliferation in Wistar isolated islets. The rate of proliferation induced by siRNA was expectedly lower than that achieved by Leucettinib-92. Indeed, the efficacy of siRNA in primary β cells is often suboptimal because of moderate transfection efficiency. Moreover, as indicated above, inhibition of other kinases such as DYRK1B by Leucettinib-92 may also contribute to the higher rate of β cell proliferation induced by Leucettinib-92 compared with that induced by anti-DYRK1A siRNAs.

In the present study, we tested the effect of Leucettinib-92 only in rat's β cells. But one should keep in mind that the response of human *versus* rodent β cells to mitogenic stimuli can be very different [42]. The lack of data with human β cells is a limitation of the present study. Further experiments using human β cells are needed, in order to assess the translational potential of our findings for the treatment of diabetes.

The present and other studies [7,14,15,17] offer the perspective of using DYRK1A inhibitors as a means to expand the β cell mass *ex vivo* prior to transplantation into type 1 diabetic patients.

While the growth-promoting effect of DYRK1A inhibitors in human and rodent's primary β cells has been widely documented in isolated islets, there are only few studies addressing a potential antidiabetic effect of DYRK1A inhibitors in T2D.

To address this question, we used a preclinical model of T2D, the Goto-Kakizaki (GK) rat, and assessed the impact of DYRK1A inhibition on the *in situ* regeneration of pancreatic β cells.

The GK rat is a spontaneous model of T2D which displays large similarities with the human disease [43]. This model is characterized by an early β cell deficiency, which is persistent throughout life [31,43].

Until weaning, GK rats are normoglycemic. At this stage, GK rats can be described as “prediabetic”. Shortly after this stage, hyperglycemia develops and overt diabetes settles at the age of 3 months and remains sustained throughout life [4,44]. The progression of prediabetic phase to the state of overt diabetes is one of the similarities between the GK model and the human T2D. The involvement of several genes, that resembles the polygenic basis for disease in the majority of type 2 diabetic patients [44,45] is another advantage of this rat model of T2D. Importantly, GK islets show a number of defects related to impairment of insulin secretion and the β cell mass, which are similar to those found in patients with T2D (for review see [[45], [46], [47]]).

These similarities make the GK model a particularly useful tool for the investigation of the potential growth promoting effects of new drugs aimed at the restoration of the functional β cell mass.

In our study, we used male GK rats because the diabetic phenotype in the GK model is more pronounced in male individuals compared to females [48,49].

Because the aim of our study was to test the glucose lowering effects of Leucettinib-92, we reasoned that a stronger diabetic phenotype would be more suitable for the investigation of anti-diabetic effects of this DYRK1A inhibitor. However, sexual dimorphism is particularly prominent in metabolic disorders [50], and further investigations are needed to extend this study to female GK rats, in order to determine the potential sex-specific effects of DYRK1A inhibitors on the remission of diabetic status.

We first assessed the effect of a short-term *in vivo* treatment with Leucettinib-92 in prediabetic GK rats and showed that a 5-day daily treatment induced β cell proliferation and increased β cell mass.

More importantly, despite the discontinuation of the treatment, the physiological benefit of increased β cell mas was preserved, leading to a significant improvement of the glycemic control over the 5 following weeks without treatment. These results indicate that regenerated β cells were stable and fully functional and contributed to the long-term regulation of glucose metabolism in Leucettinib-92-treated GK rats.

Of note, the 5-day treatment with Leucettinib-92 had no effect on the α cell mass. Recently, Karakose et al. reported that harmine induces the cycling of human α cells, that may ultimately serve as β cell progenitors [51]. Although reports by Collombat et al. also proposed this interesting hypothesis in rodent's pancreas more than a decade ago [52], the possibility of inducing transdifferentiation of α cells to β cells by pharmacological agents remains controversial [53,54]. We assessed α cell proliferation *ex vivo* in isolated islets treated with Leucettinib-92 and found no increase of glucagon-positive cell proliferation (data not shown). Therefore, in the context of our study, the possibility that increased α cell proliferation and their subsequent transdifferentiation into β cells, could contribute to the increase of the β cell mass is unlikely.

To date, only a few studies have investigated the effects of DYRK1A inhibition *in vivo*, in the context of T2D in rodents. Two elegant studies have reported that in a model of diphteria toxin A-induced β cell destruction, aminopyrazines-based DYRK1A inhibitors (twice daily for 14 days, oral administration, 50 mg/kg), induced β cell proliferation and improved the glycemic status of mice [15]. Also, inhibition of DYRK1A by harmine (daily i.p. injection, 10 mg/kg, for 7 or 14 days) induced β cell proliferation and partial recovery of the β cell mass in 60% pancreatectomized mice [14]. However, while these reports provided the proof-of-concept that DYRK1A inhibition can induce β cell proliferation *in vivo*, animal models used in these studies were not relevant to the pathophysiology of human T2D.

In this study, the first set of experiments showing a positive effect of DYRK1A inhibition in the prevention of diabetes in GK rat prompted us to investigate whether DYRK1A inhibitors can contribute to the remission of diabetes in overtly hyperglycemic rats. Of importance, we showed that the long-term chronic treatment (8 weeks, daily i.p. injection) with a low dose (0.05 mg/kg) of Leucettinib-92 did not cause any deleterious effects (no weight loss, no adverse anatomical or behavioral effects), confirming the innocuity of Leucettinib-92. The treatment led to a significant increase of both pancreatic β cell mass and insulin content. The increased insulin content could be due to a higher number of β cells within the pancreas, and also additionally, to enhanced insulin biosynthesis within individual β cells. It has been reported that beside regulating genes involved in β cell proliferation [55], NFAT also binds to the insulin promotor gene and increases its activity, leading to enhanced insulin biosynthesis [56,57]. Therefore, Leucettinib-92, in addition to its proliferation-stimulating effect, may stimulate insulin biosynthesis in β cells and help restore physiological levels of insulin within the pancreas.

Importantly, the increased β cell mass and insulin content were associated with a very significant improvement of the glycemic status in Leucettinib-92treated GK rats, as reflected by the steady reduction of random-fed morning glycemia. At the end of the treatment period, blood glucose levels of Leucettinib-92 treated GK rats were nearly 60% of the value recorded for the vehicle-treated GK rats. Also, HbA1c, used for glycemic control assessment in people with diabetes, was improved in the Leucettinib-92-treated group and the fasting blood glucose levels were significantly reduced. Furthermore, while insulin sensitivity of GK rats was only slightly affected by Leucettinib-92 treatment, GK rats treated with this compound showed improved glucose tolerance, which was associated with an enhancement of insulin secretion measured in plasma samples collected during ipGTT. These results suggest that regenerated β cells are fully functional and exhibit a glucose-regulated insulin secretion profile contrasting with vehicle-treated GK rats which display a profoundly impaired insulin secretory capacity *in vivo* in response to glucose [58].

In the present study, Leucettinib-92 was administered in a systemic manner. While most of the beneficial effects reported in this work could be attributable to the partial restoration of β cell mass and secretory function, we cannot rule out possible effects of the inhibitor on other organs involved in the regulation of whole-body glucose homeostasis. Leucettinib-92 could act directly in the liver, adipose tissue, muscle or enteroendocrine system and modulate their activities. Interestingly, we found that *glucokinase* and *ChREBP* were upregulated in the liver of GK rats treated with Leucettinib-92. Glucokinase in the primary enzyme responsible for glucose metabolism in hepatocytes and its increased expression in LCTB-92 group suggests an improvement of hepatocyte's glucose sensing and therefore improved glucose metabolism. ChREBP expression was also increased in the liver of Leucettinib-92 group. Although the exact implication of ChREBP in glucose and lipid metabolism in the liver is less clear than in the adipose tissue, it is admitted that, at least in in rodents, ChREBP, by limiting toxicity related to the accumulation of deleterious fatty acids, could be an important player of hepatic insulin sensitivity [59,60].

Deeper investigation, especially the measurement of the expression of these factors at protein level are needed to clarify the potential impact of Leucettinib-92 treatment on hepatic insulin sensitivity.

Moreover, Leucettinib-92 crosses the blood–brain barrier and may affect the central nervous system in different areas including the hypothalamus. Hypothalamus is a key player of regulation of glucose and energy homeostasis [61,62]. Activation of neurons in certain hypothalamic areas stimulates insulin secretion and controls the metabolic function of the liver through the autonomic nervous system [63]. Moreover, an ongoing work in our laboratory shows that DYRK1A is overexpressed in the hypothalamus of GK rats (unpublished data). Therefore, we can hypothesize that DYRK1A inhibition in the brain could positively impact β cell and hepatic functions that are disturbed in GK rats, and thus contribute to a better glycemic control.

Collectively, our results show that not only the inhibition of DYRK1A at the prediabetic stage can prevent the development of overt hyperglycemia, but also that it can help reduce established hyperglycemia in diabetic GK rat. To the best of our knowledge, this is the first report on the anti-diabetic effect of long-term treatment with a DYRK1A inhibitor, in a proper model of human T2D.

Despite the relevance of the use of DYRK1A inhibitors for the stimulation of β cell proliferation in the context of diabetes, the potential tumorigenic risk of DYRK1A inhibitors warrants caution. DYRK1A is extremely dosage dependent and its inhibition may indeed raise concern about possible unwanted cell proliferation. Interestingly, epidemiological and experimental evidences in recent years revealed that this enzyme has both tumor suppressor and oncogenic activities and support the idea that the final readout of overexpression or inhibition of DYRK1A depends strongly on the cellular context [33,64,65]. These aspects need to be carefully considered for the future use of DYRK1A inhibitors in humans.

Today, a number of low molecular weight DYRK1A inhibiting drugs are available [22]. However, only few of them meet the clinical requirements for their therapeutic use in humans. Cirtuvivint is in phase 1 in oncology, Lorecivivint in phase 3 for osteoarthritis [66]. A member of the Leucettinib family, Leucettinib-21, is currently in phase I clinical trial as a possible treatment of cognitive disorders associated with Down syndrome and Alzheimer's disease [41,66]. Our results with Leucettinib-92, which belongs to the same chemical family as Leucettinib-21, are encouraging and suggest that this class of DYRK1A inhibitors could be envisioned as drug candidates for the treatment of metabolic diseases.

Further pre-clinical and clinical investigations are warranted to confirm the potential of Leucettinibs as a regenerative strategy for the treatment of diabetes.

## CRediT authorship contribution statement

**Romane Bertrand:** Data curation, Methodology, Validation, Visualization, Conceptualization. **Stefania Tolu:** Investigation, Methodology. **Delphine Picot:** Methodology. **Cécile Tourrel-Cuzin:** Methodology. **Ayoub Ouahab:** Methodology. **Julien Dairou:** Investigation, Methodology. **Emmanuel Deau:** Investigation, Methodology. **Mattias F. Lindberg:** Investigation, Methodology. **Laurent Meijer:** Conceptualization, Funding acquisition, Investigation, Visualization, Writing – review & editing. **Jamileh Movassat:** Conceptualization, Formal analysis, Funding acquisition, Investigation, Methodology, Project administration, Supervision, Validation, Visualization, Writing – original draft, Writing – review & editing. **Benjamin Uzan:** Conceptualization, Investigation, Methodology, Supervision, Validation, Writing – review & editing.

## Funding

This research was supported by grants from the “*Agence Nationale* *de* *la Recherche” (ANR)* (grant KINHIB-DIAB*) and the European Union's Horizon 2020 research and innovation program* EUROSTARS grant (T2DiaCURE) to JM and LM. JM work is supported by Université Paris Cité and the Centre National de Recherche Scientifique (CNRS).

LM work is supported by the “*Fondation Jérôme Lejeune*”, the ANR (DYRK-DOWN, TRANSBIOROYAL) and France 2030 - BpiFrance (i-Nov vague 9, Leucettinib-21 project). This project has received funding from *the European Union's Horizon 2020 research and innovation program* under grant agreement No 848077 (GO-DS21) and the *European Innovation Council Accelerator* (EIC) *Accelerator Program* (DOWN-AUTONOMY project, 190138295). Views and opinions expressed here are those of the authors only and do not necessarily reflect those of the European Union which cannot be held responsible for the information it contains.

## Declaration of competing interest

Laurent Meijer is a founder of Perha Pharmaceuticals. Emmanuel Deau and Mattias F. Lindberg and Laurent Meijer are co-inventors in the Leucettinibs patents.

## Data Availability

Data will be made available on request.
